# Chromosomal Aberrations in *In-Vitro* Matured Oocytes Influence Implantation and Ongoing Pregnancy Rates in a Mouse Model Undergoing Intracytoplasmic Sperm Injection

**DOI:** 10.1371/journal.pone.0103347

**Published:** 2014-07-24

**Authors:** Min Li, Hong-Cui Zhao, Rong Li, Yang Yu, Jie Qiao

**Affiliations:** Center of Reproductive Medicine, Department of Obstetrics and Gynecology, Peking University Third Hospital, Beijing, China; Institute of Zoology, Chinese Academy of Sciences, China

## Abstract

Implantation failure and early pregnancy loss have been reported to be closely related to the quality of mammalian oocytes; however, the pregnant outcome of embryos from *in-vitro* matured (IVM) oocytes remains unknown. In this study we examined spindle assembly and chromosome segregation during differentiation, and the duration of IVM of mouse oocytes. The resulting implantation and pregnancy outcomes were analyzed to clarify the relationship between the spindle and chromosomes of IVM oocytes and implantation and early pregnancy. Cumulus-enclosed germinal vesicle oocytes were collected and randomly cultured in IVM medium with different IVM durations. One part of IVM oocytes were analyzed the spindle and chromosome morphology by immunofluorescence method, and the other part of them were fertilized by intracytoplasmic sperm injection. The resulting embryos were transferred into pseudo-pregnant female mice, and the post-implantation and full term development was observed. The chromosome aberrations and incorrect spindle assembly seems not affect the early development and blastocyst cell number derived from IVM oocytes, however the development potential of the resulting embryos after implantation were significant decreased with the ratio increasing of chromosome aberrations and incorrect spindle assembly. Accordingly, the full-term development was also decreased. In conclusion, the present study showed the spindle assembly of *in vitro*-matured oocytes was one of the most important factors that affected the implantation and ongoing pregnancy rates of IVM oocytes, and the improvement by an appropriate duration of maturation *in vitro* will enhance the post-implantation development potential of the resulting embryos, and decrease implantation failure and early pregnancy loss.

## Introduction

Assisted reproductive techniques (ART) have resulted in tremendous benefits to infertile couples since Louise Brown, the first tube baby, was born in 1978 [1]. The gonadotrophins used in the process of ovarian stimulation may induce side effects in some women, such as ovarian hyperstimulation syndrome (OHSS); however, the long-term effects of hormone stimulation remain unknown. Gonadotrophin stimulation is not always effective in stimulating follicle growth and maturation for women in ART cycles, such as poor ovarian response to gonadotropin stimulation, premature ovarian failure (POF) symptoms, and polycystic ovarian syndrome (PCOS).

Recently, cases involving *in vitro* maturation (IVM) combined with *in vitro* fertilization (IVF) have been widely reported, which has provided a potential way to resolve the issues associated with gonadotrophin stimulation [2]. The first successful clinical case using IVM oocytes was reported by Cha et al., who successfully transferred fertilized embryos derived from *in vitro*-matured oocytes into a woman with POF [3]. The transfer resulted in healthy triplet girls, which suggests that the *in vitro*-matured oocytes have the potential to develop from fertilized embryos into fetuses. Subsequently, Buckett et al. reported that a woman with PCOS became pregnant using IVM/IVF technology [4]. Despite these case reports, IVM has not been accepted as mainstream treatment in ART. After an IVM procedure, the nuclear maturation, fertilization, and cleavage rates are acceptable, but the further developmental capacity of these oocytes appears to be compromised [5]. Some studies have indicated that the higher pregnancy loss rate appears to be related to the underlying etiology for infertility (e.g., PCOS) rather than the IVM procedure [6]; however Suikkari et al. reviewed the pregnancy results of IVM cycles by some centers and suggested that at present, the most important reason for the infrequent use of IVM in most clinics is the lower pregnancy rate compared with conventional IVF/ICSI [7]. Although the clinical pregnancy rates per embryo transfer are very good, the clinical pregnancy rates per embryo transfer are still lower per oocyte collection compared with IVF/ICSI. IVM embryos have an overall probability of implantation between 7% and 12%. Indeed, one group reported higher implantation rates, but higher pregnancy loss rates after IVM [8]. To compensate for this poor embryo quality, some groups have transferred ≥3 embryos in IVM cycles to achieve acceptable pregnancy outcomes [9], [10].

The decreased developmental potential after implantation of IVM embryos has been attributed in part to aneuploidy in proximity to the spindle organization and chromosome assembly [11], therefore it is important to study the relationship between spindle and chromosome alignment and IVM embryo development, especially post-implantation to improve and optimize the IVM system and efficiency. In a clinical study, Madaschi et al. found that the selection of embryos based on the zona pellucida and meiotic spindle imaging can significantly improve implantation and pregnancy rates, which suggested the close relationship between the meiotic spindle and implantation [12]. The abnormal spindle in IVM oocytes compared to *in vivo*-matured (IVO) oocytes has been reported in some studies. Li et al. reported that IVM oocytes had a higher frequency of abnormal meiotic spindle and chromosomal alignment morphology than IVO oocytes, and suggested that IVM can have deleterious effects on the organization of the meiotic spindle and chromosome alignment of human oocytes [13]. Indeed, IVM is a potential factor to reduce the development potential, and similar results were generated in a study involving mouse IVM and IVO oocytes [14]. Due to spindle dynamics during mammalian oogenesis and oocyte maturation, and the environmental perturbations that may result in defective chromosomal partitioning during meiosis, it is essential to mimic the hormonal milieu in the ovary *in vitro* to improve spindle formation and increase IVM efficiency. Various strategies have been designed for improving cytoplasmic maturation. A study involving the composition of matured medium has shown that small molecular chemicals, such as cAMP [15], serum [16], growth factors [17], and PDE3 inhibitor [18], will improve cytoplasmic maturation; however, we recently found the developmental potential of IVM oocytes after parthenogenetic activation could be significantly improved by adjusting the maturation time [19]. The studies did not illuminate post-implantation development, therefore acknowledgment of the correlation between the spindle assembly of IVM oocytes with different maturation times and post-implantation development will be helpful in improving and optimizing IVM efficiency, and facilitating the IVM application in the clinical setting.

The present study was undertaken to evaluate the spindle and chromosome configurations of IVM oocytes with different maturation times using a mouse model, and the outcome of the resulting fertilized embryos in the pre- and post-implantation stages.

## Materials and Methods

The present study was approved by the Institutional Review Board of Peking University Third Hospital. All chemicals and reagents were obtained from Sigma (St. Louis, MO, USA) unless noted otherwise.

### Experimental design

In this study we determined the relationship between chromosome configuration and embryo developmental potential in IVM oocytes. In experiment 1, immature oocytes were collected and matured *in vitro*. All of the matured oocytes were collected at 18 h (group 1), and some portions of the oocytes were culture for an additional 2 and 4 h (groups 2 and 3, respectively). The dynamics of α-tubulin and chromosomes were analyzed using an immunofluorescence method. In experiment 2, the IVM oocytes were fertilized using the intracytoplasmic sperm injection (ICSI) method, and the developmental efficiency in the pre-implantation stage were compared with the fertilized embryos from IVO oocytes. In experiment 3, the embryos were transferred into the uterus of pseudo-pregnant mice, and the fetuses were dissected on days 6.5 and 12.5, respectively. The implantation and pregnant loss rates were analyzed and representative morphologies of the fetuses are shown.

### Animals and oocyte collection

All experiments were performed using 8–10-wk-old mice (ICR strain, from Vital River Laboratories). Animals were handled according to the Guide for Care and Use of Laboratory Animals of Peking University, and the mice were fed under constant temperature and relative humidity conditions; food and water were provided *ad libitum*. Female mice were superovulated by the intraperitoneal injection of 10 IU of equine Chronic Gonadotropin (Hua Fu Biotechnology Company, Tianjin, China), followed by 10 IU of human Chorionic Gonadotropin (Hua Fu Biotechnology Company, Tianjin, China) 48 h later. IVO MII oocytes were recovered 14–16 h after hCG injection from the ampullae of the oviducts. The cumulus cells were freed by treatment with 0.1% hyaluronidase in G-MOPES (Vitrolife, Gothenburg, Sweden). The denuded oocytes were rinsed at least 3 times and cultured under oil in groups of 20–30 to the blastocyst stage in 50- µl drops of potassium simplex optimization medium with amino acids (KSOMaa; Chemicon-. Millipore, Billerica MA). Immature oocytes were collected by puncturing visible antral follicles on the ovarian surfaces 2∼4 h after hCG injection. GV stage oocytes with an intact vestment of cumulus cells were collected and cultured in matured medium under the same culture conditions.

### IVM

Immature GV oocytes were matured *in vitro* with KSOMaa medium, including 5% fetal bovine serum and 75 mIU/ml of recombinant human FSH. The extrusion of the first polar body was used as the criterion for nuclear maturation of GV stage oocytes. The matured oocytes were selected after 18 h of culture; 1 portion of the matured oocytes were immediately used in IVF/ICSI, and the other portion of oocytes were cultured in maturation medium for an additional 4 h before ICSI.

### IVF and ICSI

For ICSI, the sperm collected from the cauda epididymis of 8–10-wk-old ICR male mice were washed 3 times with injection buffer (75 mmol/L KCl and 20 mmol/L HEPES [pH 7.0]), then treated with buffer containing 12% polyvinylpyrrolidone. The active sperm with normal morphology were selected to be injected into the oocyte cytoplasm assisted with a Piezo micropipette-driving unit (Prime Tech Ltd., Ibaraki, Japan), as previously described by Kimura and Yanagimachi [20].

Successful fertilization was assessed by the extrusion of the second polar body and two visible pronuclei in oocytes. The *in vitro* development of fertilized embryos was assessed by monitoring progression every 24 h until blastocyst stage on day 4.

### Embryo culture and embryo transfer

For embryo culture, groups of 20–30 fertilized embryos were cultured in KSOMaa medium at 37°C under 5% CO_2_ and 95% humidity.

For embryo transfer, estrus female ICR mice were mated with vasectomized male ICR mice, then the vaginal plug was checked in the morning following copulation, thus indicating successful mating. The fertilized embryos at the blastocyst stage were transferred into the uteri of pseudo-pregnant CD-1 females 2.5 d post-coitus and delivered naturally on day 17.5.

### Analysis of post-implantation embryos

The pregnant female mice were sacrificed by cervical dislocation at 6.5 and 12.5 days post-coitus. The fetuses were gently separated from the uterine wall, and washed in 0.9% NaCl 3 times. The fetuses was dissected from the fetal sacs, and the morphologies were recorded by camera.

### Immunofluorescence analysis

The method of immunofluorescence manipulation was referenced in our previous study [21]. The mouse IVM oocytes were first rinsed and fixed in 4% paraformaldehyde for 20 min, permeabilized with 0.2% Triton X-100 for 30 min, and blocked in 3% BSA in PBS for 2 h at room temperature. Incubation was carried out overnight at 4°C with primary antibody (1∶500, ab64503, Abcam, UK). After rinsing, the embryos were incubated under the same conditions with fluorescein isothiocyanate (FITC)-conjugated secondary antibody for 2 h. The nuclear status of embryos was evaluated by staining with 10 µg/mL of propidium iodide for 10 min. Finally, the embryos were mounted on glass slides and examined with a confocal laser scanning microscope (LSM710 Carl Zeiss, Oberkochen, Germany).

### Blastocyst counting

Differential staining of the inner cell mass (ICM) and trophoblastic ectoderm (TE) cells of blastocysts was performed, as referenced in our previous study [22]. Well-expanded blastocysts (120 h after HCG) were rinsed in CZB culture medium, and the embryos were placed in rabbit anti-mouse whole serum for 30 min. After treatment, the embryos were rinsed 3 times in CZB culture medium, and placed in mouse complement containing 10 mg/mL of propidiumiodide (PI) and 10 mg/mL of bisbenzimide (Hoechst 33342) for 1 h. After brief rinsing in CZB culture medium, the embryos were mounted between a slide and coverslip and examined with ultraviolet light using a Nikon fluorescence microscope.

### Real-time Quantitative PCR (qPCR)

Gene expression levels in in-vitro matured oocytes with different culture duration and fresh oocytes were determined for 2 candidate genes, including Gdf9 and Bmp15. Real-time qPCR was performed using the ABI 7500 Real-time PCR system (Applied Biosystems, Forest City, CA). PCR procedure was referred to our previous study [23]. To minimize the errors, at least 3 times experiments were performed for each sample, and for one time experiment, 50 oocytes were used. Primer sequences were shown in Table S1.

### Statistical analysis

Pre-implantation development and post-implantation dissection data were analyzed by one-way ANOVA using SPSS 13.0. Statistical significance was accepted at a P<0.05.

## Results

### 1 Spindle configuration at different maturation times

**Figure 1:**
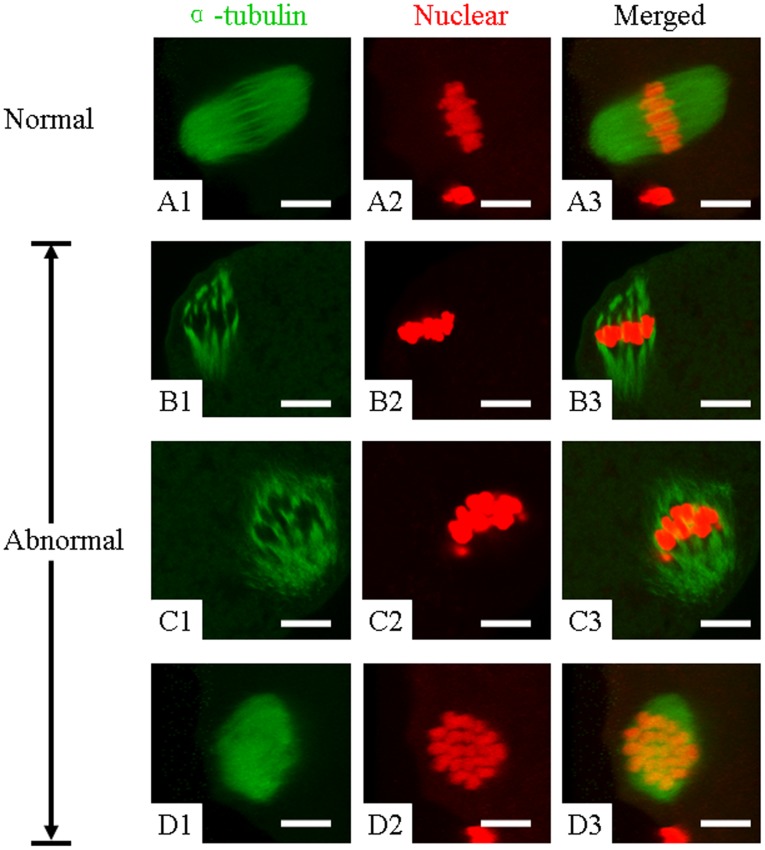
Representative imaging of four distributions and organization of microtubules and spindles in mouse IVM oocytes from maturation cultures of different duration were revealed by confocal microscopy. (A1–3) Normal spindle of rabbit oocyte with chromosomes arrayed at the metaphase plate. (B1–3 and C1–3) Abnormal spindle with disrupted microtubule bundles. (D1–3) Abnormal spindle with dispersed chromosomes. Column 1 shows the microtubule morphology by α-tubulin staining, Column 2 shows the nuclear morphology by Propidium iodide and Column 3 shows the merged images of spindle. Bar  = 7.5 µm

To test the spindle assembly, an immunofluorescence method was performed. Results of the spindle and chromosome configurations were obtained in 72, 68, 63, and 66 oocytes from groups 1, 2, and 3, and the control group, respectively. Representative images of oocytes in each group are shown in Figure 1. The spindle structure was located in all cases at the periphery of the oocyte, and oriented perpendicular to the plasma membrane. The normal morphologic spindle and chromosome assembly have specific characteristics, including chromosomes located exclusively on the equatorial plate and a barrel-shaped structure with slightly pointed poles formed by organized microtubules (Figure 1A). Abnormal chromosome assembly and spindle configurations were observed with three main forms. Abnormalities include disorganization of microtubules, and chromosomes displaced from the plane of the metaphase plate. Details of the abnormal patterns are given in Figures 1B–D. Table 1 shows the statistical results of normal and abnormal spindle morphology and chromosome organization. In the 18-h group, the percentage of IVM oocytes with normal spindle and chromosome organization was ≤50%, whereas the percentage of IVM oocytes with abnormal metaphase spindles was 52%. In the 20- and 22-h groups, the percentage of IVM oocytes with normal spindle and chromosome organization was significantly higher than the 18-h group (p<0.05), and lower than the fresh control group (p<0.05). Accordingly, the percentage of IVM oocytes with telophase spindles was significantly lower than the 18-h group (p<0.05), but no difference from the fresh control group (p>0.05). For abnormal metaphase, there was no difference among the 3 experimental groups (p>0.05), and the percentage in the first 2 groups (18- and 20-h) was significantly higher than the fresh control group (p<0.05); however, there was no difference between the 22-h and fresh control groups (p>0.05; Table 1).

**Table 1:** Summary of α-tubulin expression and nuclear location in embryos with different maturation times.

Group	No. of oocytes	Normal Metaphase (%) *	Abnormal Metaphase (%) *
18-hour	36	17 (48.21±6.30) ^a^	19 (51.79±6.30) ^a^
20-hour	32	23 (72.141.49) ^b^	9 (27.86±1.49) ^a^
22-hour	41	32 (78.33±1.67) ^b^	9 (21.67±1.67) ^a, b^
IVO	28	26 (93.06±3.68) ^c^	2 (6.94±3.68) ^b^

a–c Values with different superscripts in the same column are significantly different (*P*<0.05).

Each experiment was repeated at least three times.

*the data was shown as mean ± S.E.M.

### 2 Oocyte maturation and early development

**Figure 2:**
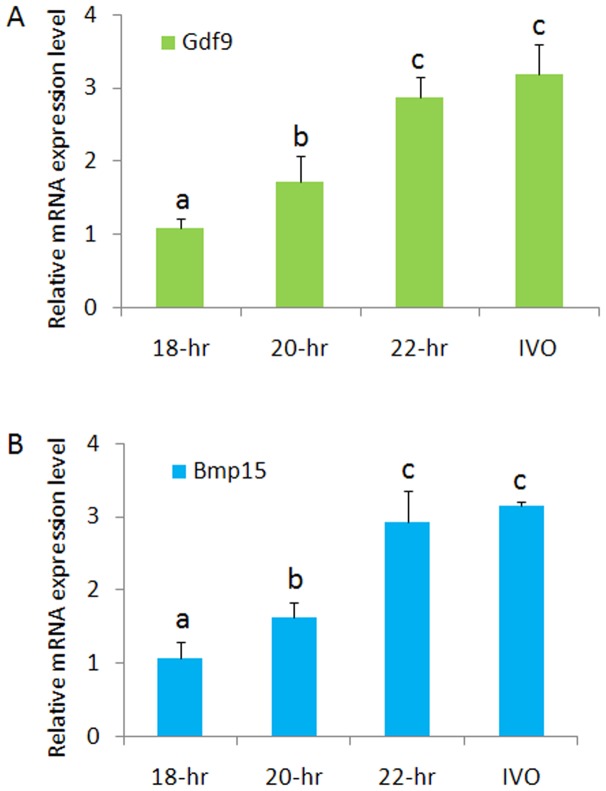
The relative mRNA expression of Gdf9 and Bmp15 in mouse IVM oocytes from maturation cultures of different duration. With the prolongation of culture duration, Gdf 9 (A) and Bmp15 (B) expression levels were increased. Different letters above the column diagram mean significant differences (P<0.05), and same letters above the column diagram mean no significant differences (P>0.05).

A total of 1072 immature oocytes were collected and cultured in IVM medium, 924 of which became matured oocytes at the MII stage. In each experimental group, there was no significant difference in the maturation efficiency (p>0.05), and >80% of the oocytes reached the MII stage after IVM culture. Gdf9 and Bmp15 genes expression levels were identified in IVM and fresh control groups. The results indicated that significantly higher expression profiling was shown in the 22-h group and fresh control group compared with that of 18-h and 20-h group, and gene expression levels of both genes in the 18-h group was significantly lower than that of the other groups (Figure 2).

Successful fertilization was assessed by male and female pronuclei formation. The fertilization efficiency was approximately 90% in the 18-h group, which was significantly lower than the other three groups (p<0.05); there was no significant difference among the three groups (p>0.05). Eighteen hours after fertilization, the 2-cell efficiency was recorded, and there was no significant difference between the 3 experimental groups and the control group (p>0.05). Forty hours after fertilization, the 8-cell efficiency was recorded. There was no difference among the 3 experimental groups (p>0.05); however, the 8-cell percentages at 18- and 22-h were significantly lower compared with the fresh control group (p<0.05). Three and one-half days after fertilization blastocyst formation was observed. The percentage of blastocyst formation in the 18-h group was significantly lower than the fresh control group (p<0.05), and the blastocyst development efficiency in the other 2 groups (20- and 22-h) was not different when compared with the 18-h group and fresh control group (p>0.05; Table 2).

**Table 2:** The early development of IVM-ICSI embryos in groups with different maturation times.

Group	No. of immature oocytes	No. of MII oocytes*	No. of Zygotes (%) *	2-cell	8-cell	Blastocyst
18-hour	314	256 (80.93±4.71) ^a^	232 (90.60±0.94) ^a^	232 (100) ^a^	190 (82.37±1.15) ^a^	138 (59.79±4.76) ^a^
20-hour	374	316 (84.96±2.01) ^a^	308 (97.81±1.35) ^b^	300 (97.27±1.22) ^a, b^	264 (85.52±3.69) ^a, b^	220 (71.54±3.67) ^a, b^
22-hour	384	352 (81.95±2.50) ^a^	340 (96.79±1.65) ^b^	328 (96.41±1.48) ^b^	272 (80.43±5.76) ^a^	224 (65.22±5.90) ^a, b^
IVO	/	240	236 (97.92±1.32) ^b^	236 (100) ^a^	224 (94.71±2.26) ^b^	182 (76.96±2.57) ^b^

a–b Values with different superscripts in the same column are significantly different (*P*<0.05).

Each experiment was repeated at least five times.

*the data was shown as mean ± S.E.M.

The number of blastocysts was calculated using a differential staining method. To count the total number of cells, there was no significant difference among the 3 experimental groups (p>0.05); however, the number of cells in the 18- and 20-h groups was significantly lower than the fresh control group (p<0.05). For ICM cell number counting, there was no significant difference among the 3 experimental groups (p>0.05), and the ICM cell number in the fresh control group was significantly higher when compared with the 3 experimental groups (p<0.05). A similar result also existed in the ratio of ICM-to-total cell number (Table 3).

**Table 3:** Cell number for fertilized embryos at blastocyst stage with different IVM timing.

Groups	Embryos examined	Total*	ICM*	ICM/Total*
18-hour	26	45.84±1.28 ^a^	11.38±5.58 ^a^	25.18±1.33 ^a^
20-hour	21	47.38±1.07 ^a^	12.00±6.25 ^a^	25.29±1.13 ^a^
22-hour	20	48.15±1.15 ^a, b^	12.10±5.32 ^a^	25.26 ± 1.07 ^a^
IVO	24	50.87 ± 1.25 ^b^	14.88 ± 5.22 ^b^	29.72 ± 1.37 ^b^

a–b Values with different superscripts in the same column are significantly different (*P*<0.05).

Each experiment was repeated at least three times.

*the data was shown as mean ± S.E.M.

### 3 Post-implantation and full-term development

**Figure 3:**
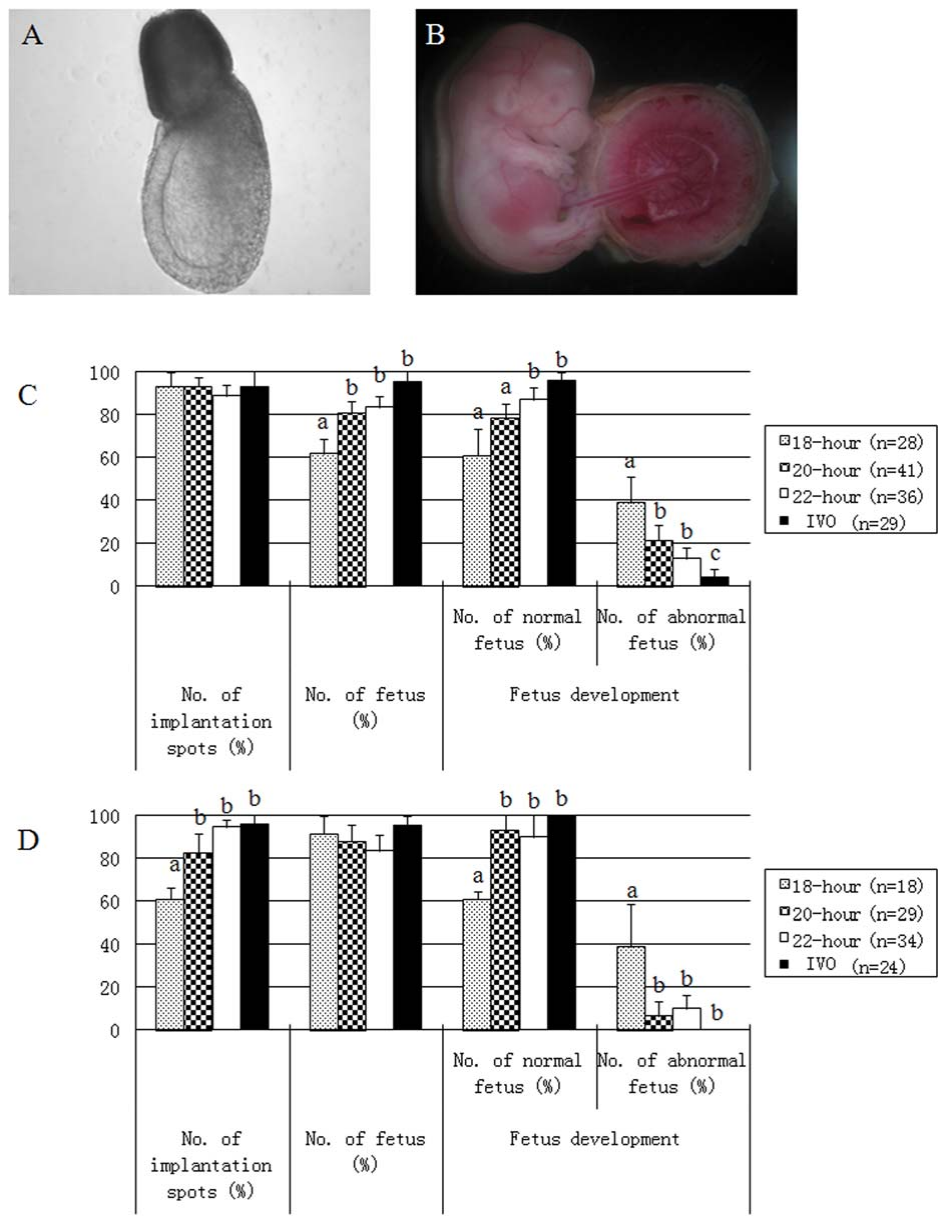
The representative fetus imaging with normal morphology on days 6.5 and 12.5 after blastocysts transfering into recipients and the assessment of post-implantation development of fertilized embryos from IVM oocytes and *in vivo*-matured oocytes.

The blastocysts were transferred into the uterus of pseudo-pregnant female mice, and the development potential of IVM embryos was studied. Approximately 6–8 blastocysts were transferred into 1 female recipient. One portion of the recipients was dissected on days 6.5 and 12.5 after embryo transfer, and the other portion of the recipients underwent cesarean section on day 17.5. Representative figures of normal fetus on days 6.5 and 12.5 are shown in Figure 3A and B. In these implantation results, the fetus are regarded as abnormality if the morphologies do not look like figure 2A and B. On day 6.5 of dissection, the implantation efficiency was no different between the 3 experimental groups and the control group (p>0.05); however, the fetal rate in the 18-h group was significantly lower than the other 2 experimental groups and the control group (p<0.05), and there was no significant difference among the 20-h, 22-h and the control groups (p>0.05; Figure 3C). The rate of normal fetuses in the 18-h group was not significantly different compared with the 20-h group (p>0.05), but significantly lower than the 22-h and control group (p<0.05). Accordingly, the rate of abnormal fetuses in the 18-h group was significantly higher than the 22-h and control groups (p<0.05). On day 12.5 after dissection, the implantation rate in the 18-h group was significantly lower than the 22-h and fresh control groups (p<0.05), but not significantly different compared with the 20-hour group (p>0.05). The fetal rate was similar in all four groups (p>0.05). In the 18-h group, the fetal rate with normal morphology was significantly lower than the other 3 groups (p<0.05), and the fetal rate with abnormal morphology was significantly higher compared the other 3 groups (p<0.05; Figure 3D).

For full-term development potential, the number of viable pups was approximately 3 in the 18-h group, which was significantly lower than the other 2 experimental groups (p<0.05), and the number of viable pups in the fresh control group was significantly higher than any experimental group (p<0.05). The weight of the placenta was not significantly different between the experimental groups and the control group (p>0.05). After 1 week, the weight of the male and female fetuses and the sex ratio were recorded; there was no difference between the experiment groups and the control group (p>0.05; Table 4).

**Table 4:** The full-term development of IVM-ICSI embryos in groups with different maturation times.

Group	Embryos transferred (recipients)	Embryos/recipient	No. of pregnant recipients to term	No. of alive pups	Weight of placenta	Weight of female fetus (1 week old)	Weight of male fetus (1 week old)	Sex ratio
18-hour	86 (12)	7.17±0.48 ^a^	6	3.00±0.37 ^a^	0.15±0.008 ^a^	3.91±0.25 ^a^	3.78±0.22 ^a^	**1.25** ^a^
20-hour	146 (20)	7.30±0.30 ^a^	10	4.50±0.34 ^b^	0.15±0.004 ^a^	3.88±0.14 ^a^	3.96±0.13 ^a^	1.25 ^a^
22-hour	130 (20)	6.50±0.40 ^a^	10	4.40±0.27 ^b^	0.14±0.006 ^a^	3.82±0.07 ^a^	3.84±0.06 ^a^	1.10 ^a^
IVO	120 (16)	7.50±0.38 ^a^	8	5.88±0.35 ^c^	0.15±0.005 ^a^	3.84±0.11 ^a^	3.97±0.12 ^a^	1.14 ^a^

a–c Values with different superscripts in the same column are significantly different (*P*<0.05).

*the data was shown as mean ± S.E.M.

## Discussion

The present study evaluated the role of chromosomal abnormalities of mouse IVM oocytes that underwent implantation failure or pregnant loss after embryo transfer. In the current study the rate of telophase oocytes in the 18-h group was significantly higher than the other two experimental groups and the control group. This finding may be caused by inappropriate maturation timing. In our previous study, we showed that an optimal duration between oocyte maturation *in vitro* and manipulation is helpful in increasing human oocyte development potential in the early developmental stage [19]. Two other groups reported similar results using fertilized embryos [24], [25]. There was no significant difference between the abnormal metaphase oocytes among the three experimental groups, but the abnormal metaphase oocytes were significant lower than the fresh control group. Chromatin organization during mammalian oogenesis and oocyte maturation has been elucidated and environmental perturbations may result in defective chromosomal partitioning during meiosis [26]. In IVM, some conditions that affect spindle organization and chromosome alignment have been reported. Roberts et al. studied the chromosome alignment in mouse metaphase I oocytes, and suggested that the FSH concentration is closely related to the aneuploidy rate when MI oocytes reach the MII stage by affecting chromosome alignment; similar results were obtained in humans [27]. Liu et al. also suggested that an increased susceptibility to meiotic errors in early stage follicles undergoing *in vitro* culture was probably relative to the chromosome aneuploidy rate in IVM oocytes [28]. Moreover, a low oxygen concentration and cooling and re-warming were also shown to induce meiotic errors in the process of oocyte maturation *in vitro* [29], [30].

Abnormal spindle organization and chromosome assembly was closely related to the subsequent development of embryos. In some studies, abnormal spindle organization and chromosome assembly acted as one of the potential predictors of oocyte quality and embryo developmental potential in human ART. Rienzi et al. reported that oocytes with a deviation of the spindle location from the position of the polar body of >90^o^ showed lower fertilization rates [31], and similar results were reported by other groups [32], [33]. Raju et al. reported that visible and retardation of the spindle was close to blastocyst formation of human fertilized embryos with ICSI [34]. Petersen et al. used meta-analysis to investigate the influence of meiotic spindle visualization in human oocytes on ICSI outcomes, and suggested that pronuclear formation, but not fertilization, of day 3 embryo scoring and blastocyst formation were significantly higher in oocytes with normal meiotic spindles compared with abnormal meiotic spindles [35]. Based on our results, the developmental potential in every developmental stage was significantly lower than the control group, including fertilization, pronuclear formation, cleavage, 8-cell stage, and blastocysts, and these results were consistent with previous human studies [35]; however, we showed that IVM oocytes had different developmental potential with a prolonged maturation duration from 18–22 h, and embryo development was also improved. In our previous study, we also showed that if we prolonged the IVM duration appropriately, human parthenogenetic embryo developmental potential was increased significantly [19]. In the other two groups, the delayed manipulation of IVM oocytes may be helpful for pronuclear formation and development before zygotic genome activation of the fertilized embryos [24], [25]. The oocytes quality was evaluated by the gene expression identification for two important genes that were Gdf9 and Bmp15. Both genes were thought as a key to determine oocyte maturation quality [36], [37]. In the present study, the expression levels were gradually increased with the prolongation of culture duration after maturation in vitro, which suggested the quality of oocytes was improved and therefore contribute to the resulted higher development potential in them.

The blastocyst quality was evaluated by a differential staining method, and the results showed a similar trend observed in developmental efficiency *in vitro*. The total cell number, the ICM cell number, and the ratio of the ICM-to-total were significantly lower in all three IVM groups compared with the control group. Unlike the development potential *in vitro*, the prolonged maturation timing was not helpful with the cell number of the resulting blastocysts. This finding may be attributed to the comparative poor intrinsic quality of IVM oocytes compared with fresh oocytes, and there are a number of reports in the literature indicating that bovine oocytes matured *in vivo* are more competent that those matured *in vitro* [38], [39], [40], [41].

In our study, the pregnant mice were dissected on days 6.5 and 12.5, respectively. In mouse primitive streak will begin to form on day 6.5, which is equivalent to 2–4 weeks after fertilization in human embryos [42], [43]. The primitive streak is one of the most important developmental stages in the early implantation process. Bilateral symmetry is established in the primitive streak, the site of gastrulation is determined, and germ layer formation is initiated. Further, important transcription factors are elaborated and signaling pathways are started that induce the cells differentiated into the three germ layers (endoderm, mesoderm, and ectoderm) in the primitive streak will give rise to all of the tissues of the adult organism. On day 12.5, the embryos reach the late developmental stage, and the important tissues and organs complete development. Based on our results for the dissection on day 6.5, the implantation efficiency is not different; however, the fetus-to-normal fetus ratio is significantly different among the three experimental groups and the control group. This indicated that the trophoblast cells in IVM embryos are normal, but the ICM cells lost the ability to form a healthy fetus. Based on our results for the dissection on day 12.5, there was a significant difference in implantation efficiency, which was due to the degenerated fetus in the early implantation stage being absorbed by the maternal uterus. Furthermore, the normal fetus number in the 18-h group was still significantly lower than in the 20- and 22-h groups and the control group, whereas there was no difference among the three groups. Thus, IVM timing plays a key role in IVM embryo development in the post-implantation stage. This conclusion is also confirmed by the results of development to term. Recently, Madaschi et al. reported that the selection of embryos based on the zona pellucida and meiotic spindle imaging can significantly improve implantation and pregnancy rates [12].

In the current study we found that the abnormal spindle and chromosome misalignment in IVM oocytes has a close relationship to embryo development, especially for post-implantation stage. One of the possible reasons is aneuploidy induced by the abnormal spindle and chromosome organization [44]. That spindle alterations may predispose oocytes to aneuploidy or maturation arrest has been demonstrated in some studies [45], [46], and the meiotic defects in the spindle assembly checkpoint contribute to high susceptibility to aneuploidy [47] and similar phenomena was found in cancer cells [48]. The limited developmental potential of IVM embryos has partly resulted in chromosome aneuploidy in recent studies. Nogueira et al. analyzed embryos after IVM maturation by investigating the nuclear status and cytogenetic constitution. A high incidence of multinuclear blastomeres and aneuploidy, suggesting abnormal cytokinesis or genetic abnormalities, was observed [49]. IVM timing was also a factor with respect to the induction of chromosome aneuploidy of IVM embryos. Zhang et al. studied the chromosome abnormality rates in human embryos obtained from *in vitro* maturation and IVF treatment cycles, and they found that the aneuploidy will be significantly increased in embryos derived from oocytes that matured 48 h after collection compared with oocytes matured 24 h or the control group [50]. Emery et al. also reported that the incidence of aneuploidy in embryos with delayed fertilization for IVM oocytes would reach approximately 80%, which was significantly higher than in the control group [51]. For implantation, Akiyama et al. concluded that aneuploidy caused in the meiosis of mouse oocytes will induce fetal loss and the litter size is significantly decreased [52]. Requena et al. suggested that the high incidence of chromosome abnormalities in embryos resulting from the IVM protocol may account for the low implantation rates [11].

The physiology index is recorded in our study. The placenta weight, neonate weight, and sex ratio were no different among all four groups. A risk evaluation using IVM oocytes has been performed in some groups. Söderström-Anttila et al. analyzed the obstetric and perinatal data were collected from all deliveries after IVM treatment during 1999–2004, and found the mean birth weight of the infants was normal, and minor developmental delay was overexpressed at 12 months, but the development of the children was normal at 2 years [53]. Buckett et al. indicated that IVM did not produce any additional risks compared with traditional IVF or ICSI treatment [54]. Shu-chi et al. evaluated the physical growth and developmental indices of IVM children with a combination priming protocol using FSH and hCG, and suggest that the offspring of IVM pregnancies did not show developmental delay during infancy and early childhood [55]. However, in the long-term risk evaluation using mouse models, Eppig et al. found that a slight reduction in pulse rate and cardiac output in the IVM mouse models, although IVM of oocytes has minimal effects on the long-term health of offspring [56]. Thus, long-term follow-up of offspring IVM pregnancies is necessary because the first IVM offspring is on 23 years old.

Mouse models were applied in the present study, and there are some differences for the oocytes between mouse and human. Much better maturation synchronization was found in mouse oocytes, and on the contrary it is difficult to determine the accurate timing for maturation. In our previous study, we have proved that prolongation of culture duration can improve the development potential in artificial activated human oocytes [19], which was accordance with our present results in mouse, however these oocytes have to be carried out of incubators continuously every two hours, which limited the application in clinic because this manuplation will harm oocyte or embryo development potential. Time-lapse technique has been applied in clinic now [57], and by this technique, it is easier to observe the oocyte maturation and embryo development without taking them out of incubator. Therefore it is probably that we can record the timing of the first polar body expelling of IVM oocytes, and fertilize them after appropriate prolongation, and promote this improvement method to be applied in clinic settings in future.

In conclusion, the present study showed a higher incidence of chromosome abnormalities in embryos resulting from the IVM timing and the close relationship to post-implantation development using mouse models. However post-implantation development is still different from the control group after optimizing IVM timing, which means there are still other factors to affect the development potential of IVM embryos. Future studies, including improvement and optimization in the IVM system and gene expression related to implantation, are needed to elucidate the mechanisms of implantation failure and pregnancy loss.

## Supporting Information

Table S1 **PCR primer sets used for PCR reaction.**
(DOCX)
