# Supplementary material for: Chromosomal Aberrations in In-Vitro Matured Oocytes Influence Implantation and Ongoing Pregnancy Rates in a Mouse Model Undergoing Intracytoplasmic Sperm Injection
Source: PLoS One. 2014 Jul 24;9(7):e103347. doi: 10.1371/journal.pone.0103347 (PMC4110001; doi:10.1371/journal.pone.0103347)
Supplement: Table S1 — PCR primer sets used for PCR reaction. (DOCX) [file pone.0103347.s001.docx]

Table S1 Primer sets used for PCR reaction

| Gene | Primer Sets (Forward 5′- 3′) | Primer Sets (Reverse 5′- 3′) |
| --- | --- | --- |
| *Gdf9* | CAAACCCAGCAGAAGTCAC | AAGAGGCAGAGTTGTTCAGAG |
| *Bmp15* | AAATGGTGAGGCTGGTAA | TGAAGTTGATGGCGGTAA |
